# A case of systemic mastocytosis mimicking POEMS syndrome: A case report

**DOI:** 10.1097/MD.0000000000028651

**Published:** 2022-02-04

**Authors:** Yanqiu Hou, Suyu Jiang, Lu Zhang, Yan Wang, Liubo Zhang, Hongyu Bao, Qingqi Meng, Xue Han, Wanru Chen, Tiantian Li, Jie Peng, Yilin Zhu, Rong Huang, Jingan Liu, Jianning Wang, Chao Fang, Xiaofeng Shi

**Affiliations:** a Second Affiliated Hospital of Nanjing Medical University, Nanjing, China; b Affiliated Hospital of Jiangsu University, Zhenjiang, China; c Department of Pharmacology, School of Basic Medicine, Tongji Medical College, Huazhong University of Science and Technology, Wuhan, China.

**Keywords:** c-Kit mutation, differential diagnosis, pathology, polyneuropathy, organomegaly, endocrinopathy, M protein, and skin changes syndrome, systemic mastocytosis

## Abstract

**Rationale:**

POEMS (polyneuropathy, organomegaly, endocrinopathy, M protein, and skin changes) syndrome is a rare and complicated disease related to multiple organs and systems. Here, we report a case of systemic mastocytosis (SM) that was misdiagnosed as a POEMS syndrome.

**Patient concerns:**

A 42-year-old man presented with skin changes, diarrhea, and limb numbness.

**Diagnoses:**

Positron emission tomography/computed tomography revealed extravascular volume overload, organomegaly, lymphadenopathy, and bone lesions with mixed lesions of osteosclerosis and osteolysis. Therefore, POEMS syndrome was suspected. Further histopathological and immunohistochemical examination of the bone marrow, lymph nodes, and gastric mucosa suggested a diagnosis of mastocytosis. The *c-Kit D816V* mutation confirmed the diagnosis of SM.

**Interventions:**

The patient received the treatment of pegylated interferon-alpha weekly and glucocorticoid daily.

**Outcomes:**

The symptoms relieved significantly.

**Lessons:**

There are many similar features between POEMS syndrome and SM, probably leading to misdiagnosis. This study analyzed the different points between them which can provide help for differentiation.

## 1 Introduction

POEMS (polyneuropathy, organomegaly, endocrinopathy, M protein, and skin changes) syndrome is a rare paraneoplastic disorder associated with underlying plasma cell dyscrasia that involves multiple organs and systems.^[1,2]^

Mastocytosis is one of the myeloproliferative neoplasms.^[3]^ The WHO classification divides mastocytosis into: cutaneous mastocytosis; systemic mastocytosis (SM): indolent SM, smoldering SM, SM with an associated hematological neoplasm, aggressive SM, and mast cell leukemia; and mast cell sarcoma.^[4,5]^ SM is a heterogeneous disease characterized by the accumulation of neoplastic mast cells in the bone marrow and other organs/tissues.^[5]^ SM also demonstrates complicated signs and/or symptoms.

SM and POEMS syndromes share similar symptoms and misdiagnosis is common. Here, we report a case of SM that was misdiagnosed as a POEMS syndrome.

## 2 Case report

**Figure 1:**
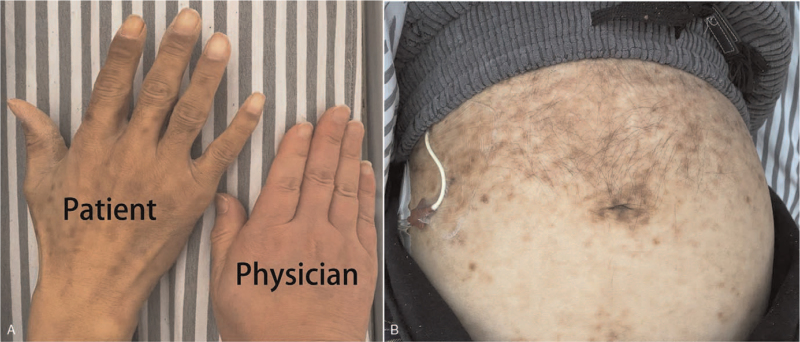
A. The skin pigmentation and white nails of patient's hand (Left). B. Multiple pigmented macules in abdominal skin.

**Figure 2:**
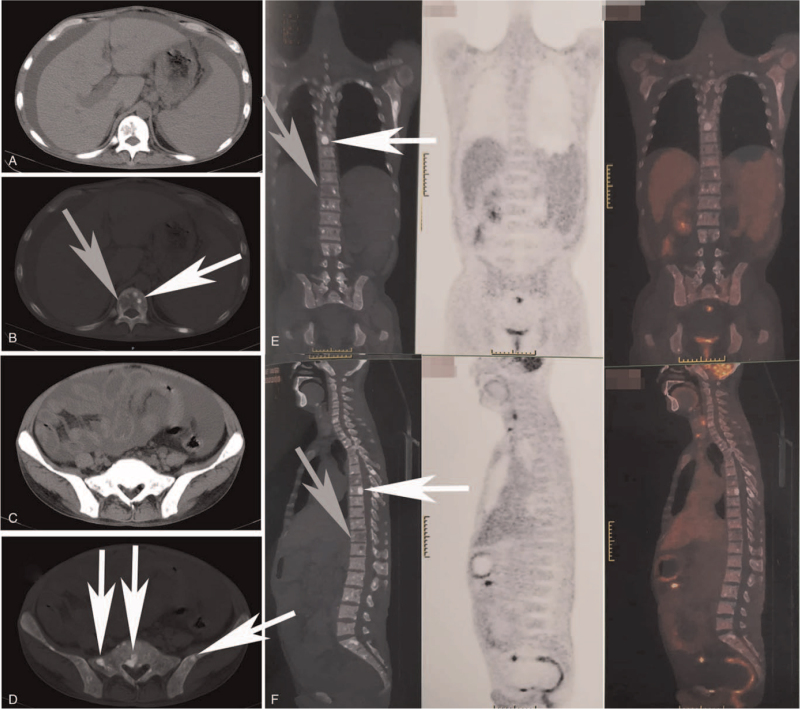
A–D. CT scan showed hepatosplenomegaly and ascites (A), mixed lesions with osteosclerosis and osteolysis in lumbar vertebrae (B), ascites and swelling bowels (C), and osteosclerosis in sacrum and ilium (D) (The white arrows direct the osteosclerosis and the grey arrows direct the osteolysis.). E and F. PET/CT showed mixed bone lesions in spinal column with normal SUV of ^18^FDG (The white arrows direct the osteosclerosis and the grey arrows direct the osteolysis.). CT = computed tomography, PET = positron emission tomography, SUV = standard uptake value.

A 42-year-old man was admitted to our hospital on January 5, 2021, complaining of diarrhea for more than 20 years and skin rash for 14 years. The rash was itchy and red but caused hyperpigmentation after scratching (Fig. 1A and B). Administration of antiallergic drugs such as loratadine, cetirizine, or ketotifen could alleviate itching. The patient gradually experienced fatigue and weakness, with weight loss and numbness in both lower limbs. Blood test showed white blood cell 4.6 × 10^9^/L (4∼10 × 10^9^/L), hemoglobin 103 g/L ↓ (110∼150 g/L), platelet count 155 × 10^9^/L (100∼300 × 10^9^/L), C-reactive protein 17.54 mg/L ↑ (0∼10 mg/L), albumin 32.1 g/L ↓ (40∼55 g/L), urea nitrogen 10.8 mmol/L ↑ (3.1∼8.8 mmol/L), creatinine 222.86 μmol/L ↑ (41∼81 μmol/L), uric acid 611.62 μmol/L ↑ (143∼339 μmol/L), triiodothyronine (T3) 3.4 pmol/L ↓ (3.5∼6.8 pmol/L), thyroxine 9.37 pmol/L ↓ (12∼22 pmol/L), and TSH 11.75 μIU/mL ↑ (0.27∼4.2 μIU/mL). Electromyography suggested demyelinating lesions of the peripheral nerves in both the lower limbs. Computed tomography (CT) scan showed splenomegaly, enlargement of retroperitoneal and bilateral inguinal lymph nodes, a large amount of effusion in the abdominal and pelvic cavity, swelling of the intestinal wall, and mixed lesions of osteosclerosis and osteolysis in the ribs, thoracolumbar vertebrae, and pelvis (Fig. 2A–D). Further, positron emission tomography/CT showed no significant increase in 18 Fluorodeoxyglucose (^18^FDG) metabolism in mixed bone lesions (Fig. 2E and F). Enlarged lymph nodes with normal standard uptake values are also shown in Figure 2E and F. The other imaging features were similar to those observed on CT.

Since polyneuropathy, organomegaly, endocrinopathy, skin changes, extravascular volume overload (edema, pleural effusion, or ascites), and osteosclerosis were present, POEMS syndrome was considered.

**Figure 3:**
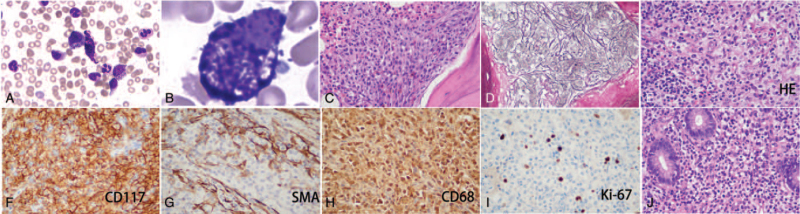
A and B. Bone marrow smear (Wright staining, arrow). C. Bone marrow biopsy (hematoxylin-eosin [HE] staining). D. Bone marrow reticulin staining. E. Biopsy from retroperitoneal lymph nodes (HE staining). F–I. Immunohistochemistry of lymph nodes. J. The biopsy from gastric mucosa (HE staining).

**Figure 4:**
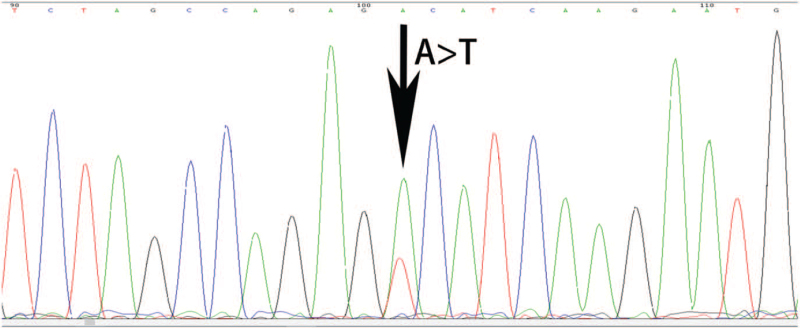
Sequencing of DNA from bone marrow mononuclear cells confirmed the *c-Kit* mutation (c2447A>T).

However, no monoclonal immunoglobulin was identified by immunofixation electrophoresis. The level of plasma vascular endothelial growth factor (VEGF) was 34.38 pg/mL (6.25–426 pg/mL). A bone marrow smear showed an increased number of spindle-shaped mast cells (Fig. 3A). Many fine eosinophilic granules were apparent in the cytoplasm of mastocytes at high-power magnification (Fig. 3B). Karyotype analysis of the bone marrow mononuclear cells revealed 46, XY [20]. A bone marrow biopsy showed characteristic focal aggregates of mast cells (Fig. 3C). Bone marrow reticulin staining increased (Fig. 3D). Immunohistochemistry of bone marrow biopsy showed local proliferation of abnormal cells (30%) with CD34+, myeloperoxidase (MPO) (myeloid cells+), CD117+, CD2+, tryptase+, chymase+, and flow cytometry of bone marrow cells showed 47% abnormal cells expressing CD117, CD13, and CD25, but did not express CD34, CD2, and CD22, suggesting mastocytosis. So further laparoscopy for retroperitoneal lymph node biopsy was performed and the result showed no Castleman-like change, but the proliferation of mast cells with CD117(+), CD68(+), Ki67 (about 10%+), SMA (partial+), CKpan(−), CD20 (B cells+), Pax-5 (B cells+), CD3 (T cells+), CD43 (T cells+), CD4 (T cells+), CD8 (T cells+), CD23 (DC cells+), CD21 (DC cells+), CD138(−), Syn(−), CgA(−), CD56(−), S-100(−), a-inhibin(−), Des(−), CD34(−), HMB45(−), MelanA(−), Dog-1(−), ALK(−), PLAP(−), and MPO(−) (Fig. 3E–I). Pathology of the gastric mucosa collected through gastroscopy showed acutely active inflammation and mastocyte infiltration (Fig. 3J). Colonic mucosa had the similar pathological change to gastric mucosa with CD117(+), CD2(+), CD20(−), CD3(−), CD43(+), CD68(+), CD38 (scattered plasma cells+), CMV(−), Ki67 (3%+), and EBER(−). An aggressive SM was considered. By sequencing of 42 genes which are related with acute myeloid leukemia/myeloproliferative neoplasms/myelodysplastic syndrome including *ASXL1, CALR, CBL, CEBPA, CSF3R, CUX1, DNMT3A, EED, ETV6, EZH2, FLT3, GATA2, GNAS, HRAS, IDH1, IDH2, IKZF1, JAK2, JAK3, KDM6A, KIT, KRAS, MPL, NF1, NOTCH1, NPM1, NRAS, PTPN11, RUNX1, SETBP1, SF3B1, SH2B3, SRSF2, STAG2, SUZ12, TET2, TP53, U2AF1, WT1, BCOR, PHF6*, and *ZRSR2*, the mutations of *c-Kit* (p.D816V, 32.35%), *DNMT3A* (p.R882H, 45.15%) were detected (Fig. 4). Therefore, the diagnosis of SM was confirmed.

Due to economic limitations, the patient refused treatment with avapritinib and midostaurin. The patient started treatment with pegylated interferon-alpha weekly and glucocorticoids daily, with significant symptom relief.

## 3 Discussion

POEMS syndrome is an underlying plasma cell dyscrasia that involves multiple organs, systems, and diseases. The diagnosis of POEMS syndrome requires meeting both mandatory major criteria (polyneuropathy and monoclonal plasma proliferation) and 1 of the 3 other major criteria (Castleman disease, sclerotic bone lesions, and VEGF elevation) and 1 of the 6 minor criteria (organomegaly, extravasular volume overload, endocrinopathy, skin changes, papilledema, and thrombocytosis/polycythemia), or other symptoms and signs (clubbing, weight loss, hyperhidrosis, pulmonary hypertension/restrictive lung disease, thrombotic diatheses, diarrhea, and low vitamin B_12_ values).^[2]^ This patient had polyneuropathy (demyelinating lesions of the peripheral nerve), sclerotic bone lesions, organomegaly, extravascular volume overload, endocrinopathy (hypothyroidism), skin changes, weight loss, and diarrhea. The future negative results of immunofixation electrophoresis ruled out a diagnosis of POEMS syndrome. Additionally, plasma VEGF levels were normal. At the same time, the biopsy of the lymph nodes did not display Castleman-like changes, but infiltration of mast cells.

The diagnosis of SM can be made when the major criterion and at least 1 minor criterion are present or when ≥3 minor criteria are present.^[5]^ The major criterion involves the multifocal infiltration of mast cells (≥15 mast cells in aggregates) in the bone marrow and/or other extracutaneous organs. Minor criteria include: the presence of atypical mast cells in the tissues. Twenty-five percents of the mast cells in the infiltrate are spindle-shaped or have atypical morphology, or >25% of all mast cells in bone marrow aspirate smears are immature or atypical; presence of activating gain-of-function point mutation in *c-Kit* D816V in neoplastic mast cells in the peripheral blood, BM, or visceral organs; aberrant expression of CD117, CD2, and/or CD25 in neoplastic mast cells; and persistently elevated serum tryptase levels (>20 ng/mL). For this patient, the major criteria and at least 2 minor criteria were fulfilled. Additionally, the abnormal cells were positive for CD43 and CD68, which may have caused misperception with histiocytes, T lymphocytes, or even blast cells. However, the lack of T-cell antigens other than CD2 or MPO helped exclude these cell types. This patient manifested an indolent form for at least 14 years, experienced an aggressive form, and finally progressed to develop mast cell leukemia.

Both POEMS syndrome and SM involve multiple organ systems. Patients often visit different doctors from different departments. The mean time from symptom onset to final diagnosis was 9 years. There are many similar features between POEMS syndrome and SM, which may lead to misdiagnoses. However, the mechanisms causing these symptoms and/or signs are different, thus causing some subtle differences between them, which can help us obtain a correct diagnosis.

Peripheral neuropathy is one of the major criteria for the diagnosis of POEMS syndrome.^[2]^ Peripheral neuropathy is a rare condition in patients with SM. To our knowledge, only 1 case of SM has been reported to have peripheral neuropathy that resolved after therapy.^[6]^ In this study, the patient had numbness in both lower limbs, which was confirmed by electromyography as demyelinating lesions of the peripheral nerve.

Osteosclerosis is one of the major criteria of POEMS syndrome and occurs in approximately 95% of POEMS syndrome patients.^[2]^ Some osteosclerosis is mixed with osteolysis, and the former has normal ^18^FDG metabolism, while the latter has hypermetabolism with an increased standard uptake value.^[7–10]^ Osteolysis is associated with hyperproliferation of plasma cells. SM, similar to POEMS syndrome, also has mixed lesions of osteolysis and osteosclerosis. Osteolytic lesions in SM can cause pathological fractures.^[11]^ While ^18^FDG uptake does not appear to be a sensitive marker of mast cell activation or proliferation, because no significant ^18^FDG uptake was observed in the most common forms of mastocytosis.^[12]^ In this patient, the mixed bone lesions showed normal ^18^FDG uptake.

The skin changes in POEMS syndrome often demonstrate themselves as hyperpigmentation, hemangioma, hypertrichosis, acrocyanosis, white nails, facial atrophy, flushing, or clubbing, probably resulting from elevated VEGF or adrenocortical insufficiency, *among others*,^[2,9]^ but no itch. Skin changes in SM are associated with itching. If local pressure is applied to the skin, individual lesions show urtication and become raised, pruritic, and erythematous, often resulting from elevated basal serum tryptase and/or histamine level.^[13]^ The patient experienced red itchy urtication and hyperpigmentation after scratching.

The extravascular volume overload is due to increased vascular permeability, which is caused by elevated VEGF in POEMS syndrome^[2]^ and by increased cytokines in SM.^[14]^ Hepatosplenomegaly results from increased vascular permeability in POEMS syndrome^[2]^ and mastocyte infiltration in SM.^[15,16]^

Diarrhea is another symptom and sign of POEMS syndrome.^[2]^ Gastrointestinal diseases and associated symptoms, including diarrhea, nausea, and vomiting, are commonly associated with SM.^[17]^ This patient had at least 14-year history of diarrhea, probably associated with elevated histamine levels.

Mutations in c-*Kit* D816V have been detected in over 80% of patients.^[5,18]^ The Kit receptor is encoded by a 21-exon containing gene located on human chromosome 4q12, which expresses a 976-amino acid protein with a molecular weight of 145 kDa. The receptor is composed of an extracellular domain, juxtamembrane domain, and a tyrosine kinase domain. Tyrosine kinase domain contains a phosphotransferase domain and ATP-binding site. The mutation of c-*Kit* D816V (NM_000222:c2447A>T/p.D816V), primarily an aspartic acid to valine substitution (D816V) in the second catalytic domain, results in enhanced survival and autonomous growth of neoplastic mast cells. Recent studies have shown that >60% of patients with advanced SM harbor somatic variants of genes other than *c-Kit*. These additional mutations affect genes encoding transcription factors, signaling molecules, epigenetic regulators, or splicing factors, resulting in shorter overall survival.^[13,19,20]^*DNMT3A* mutations are present in approximately 12% of patients with SM,^[19,20]^ suggesting a poor prognosis. Mutations in *c-Kit* and *DNMT3A* were found in this patient.

In conclusion, in this study, we compared the differences and similarities between SM and POEMS syndrome, providing hematologists with increased awareness of the 2 kinds of rare diseases.

## Author contributions

**Conceptualization:** Chao Fang, Xiaofeng Shi.

**Formal analysis:** Yanqiu Hou, Suyu Jiang, Xiaofeng Shi.

**Funding acquisition:** Xiaofeng Shi.

**Investigation:** Yanqiu Hou, Suyu Jiang, Lu Zhang, Yan Wang, Xiaofeng Shi.

**Methodology:** Wanru Chen, Tiantian Li, Jie Peng, Yilin Zhu, Rong Huang, Jingan Liu.

**Project administration:** Lu Zhang, Yan Wang, Liubo Zhang, Hongyu Bao, Qingqi Meng, Xue Han.

**Resources:** Yanqiu Hou, Suyu Jiang, Lu Zhang, Yan Wang, Jianning Wang, Xiaofeng Shi.

**Writing – original draft:** Xiaofeng Shi.

**Writing – review & editing:** Chao Fang.
